# A manifesto for coastal landscape governance: Reframing the relationship between coastal and landscape governance

**DOI:** 10.1007/s13280-024-02040-5

**Published:** 2024-06-01

**Authors:** Carla Gonçalves, Paulo Pinho

**Affiliations:** CITTA - Research Centre for Territory, Transports and Environment, Department of Civil Engineering, Faculty of Engineering, University of Porto, Rua Dr. Roberto Frias s/n, 4200-465 Porto, Portugal

**Keywords:** Climate crisis, Integrated coastal zone management, Landscape approaches, Landscape sustainability science, Socio-ecological systems

## Abstract

There is an urgent demand for substantial reforms in the governance of coastal regions. Recent research advocates for a transformative shift in European coastal governance system towards a landscape governance approach. This perspective, informed by a narrative literature review on coastal and landscape governance, explores the potential value of coastal landscape governance, drawing on the Council of Europe Landscape Convention. Our results, presented in the form of a manifesto, underscore the need to move beyond political administrative boundaries and address all coastal landscapes as socio-ecological systems. It emphasises the necessity for the State to recognise them as a public and common good, establishing a specific governance arena with dedicated actors and institutions. The manifesto also advocates for landscape justice through knowledge co production, urging transformative change and landscape based regional design to envision alternative futures. Additionally, it calls for regionalising coastal landscape governance and invites scholars from other transdisciplinary and interdisciplinary perspectives to contribute to this research agenda.

## Introduction

The sustainable governance of coastal regions continues to be considered a relevant challenge in the twenty-first century (Kelly et al. 2019, p. 1). Despite scientific and political efforts to implement an Integrated Coastal Zone Management approach in many coastal countries, there is an evident need for large-scale reform in coastal governance (Pittman and Armitage 2016). Researchers are calling for an evolved governance paradigm that embraces the boundaries, dynamics, and specific challenges of the coastal socio-ecological system (Schlüter et al. 2020; Van Assche et al. 2020).

In landscape research, the debate explores the advantages of using the landscape as a medium for governance at different scales, in addition to its role as an object of planning and management (Van Oosten et al. 2021; Van Rooij et al. 2021). Since 2007, following the systematic conceptualisation of landscape governance provided by Görg (2007), there has been a growing debate regarding both its conceptualisation and operationalisation. However, the body of knowledge on coastal landscapes and their governance is still relatively small (Döring and Ratter 2018). Nevertheless, recent research on the evolutionary governance of the Portuguese coastal region since 1950 (Gonçalves and Pinho 2024) has demonstrated the instrumental role that landscape thinking has played in coastal governance. The study’s findings highlight the correlation between the level of landscape integration within the governance system and the protection of coastal regions. The authors advocate for a paradigm shift in European coastal governance towards a landscape-governance approach, emphasising the urgency for substantial reforms in the governance of coastal regions (Kelly et al. 2019) and drawing upon the principles outlined in the Council of Europe Landscape Convention (Council of Europe 2000, 2016). The Convention was introduced in 2000 by the Council of Europe with the aim of promoting landscape protection, management and planning, as well as facilitating European cooperation on landscape issues (Council of Europe 2000, 2016).

The proposed reform of coastal governance towards coastal landscape governance, as argued by Gonçalves and Pinho (2024), aims to establish a holistic governance framework that prioritises the so-called *coastal condition* [what makes coastal areas unique] (Van Assche et al. 2020, p. 3). Besides acknowledging the unique characteristics of coastal areas, it also recognises the dynamics of the coastal socio-ecological system and gives prominence to the voices of coastal communities. This approach builds on the conceptualisation of landscape governance as a new paradigm for the governance of coastal regions. While the potential implications of this paradigm shift remain unexplored, their arguments align with ongoing discussions of coastal governance (Pittman and Armitage 2016; Schlüter et al. 2020), of landscape governance (Görg 2007; Kozar et al. 2014), and of Integrated Landscape Approaches (Reed et al. 2020).

In this perspective article, we aim to explore the potential advantages of reorienting coastal governance towards coastal landscape governance. Through a critical examination of the broader scholarly discourse on coastal and landscape research, we discuss the theoretical value of coastal landscape governance as a catalyst for reframing the relationship between coastal and landscape governance.

**Fig. 1:**
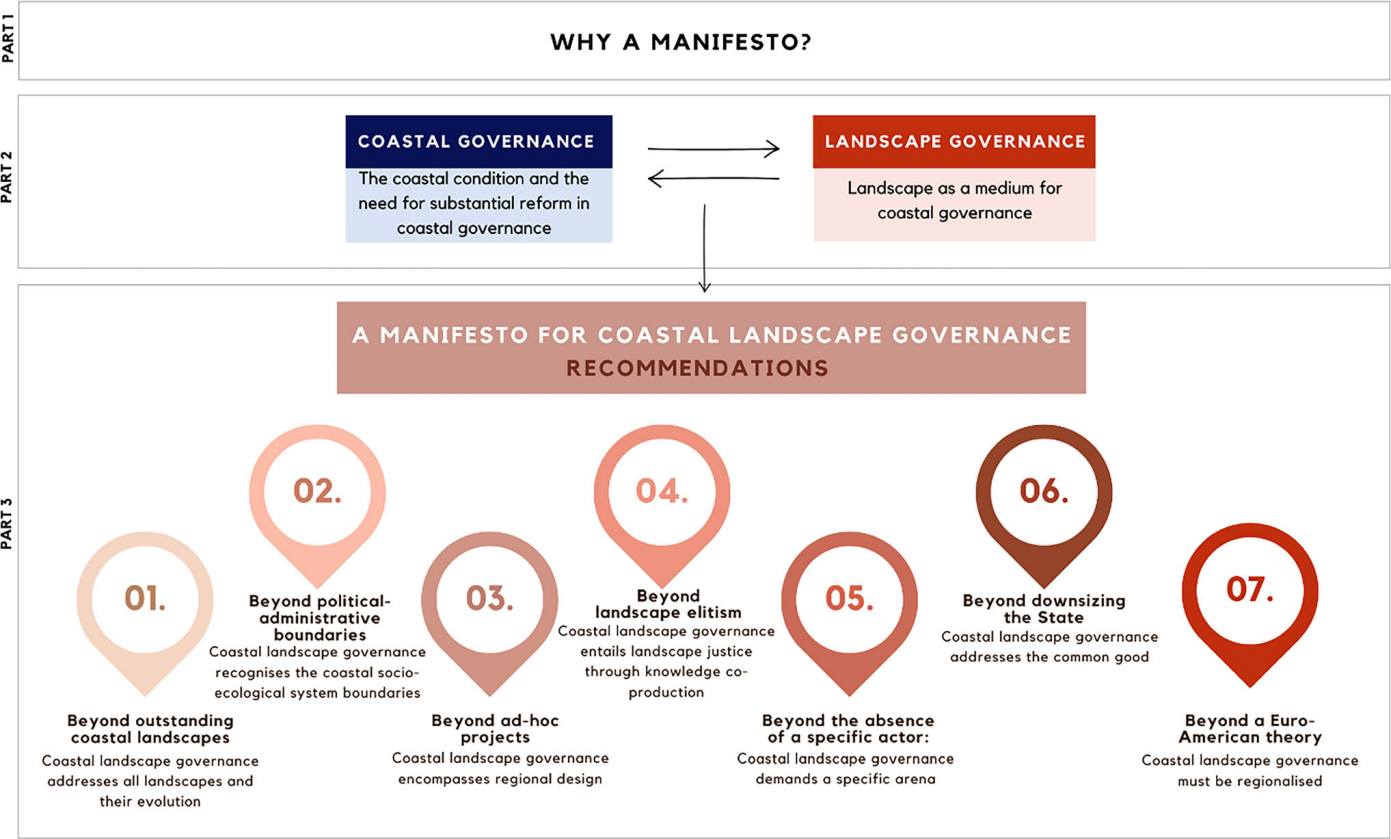
Structure of the perspective paper and the seven recommendations of the manifesto for coastal landscape governance

This article, presented in the form of a manifesto, is structured as follows (Fig. 1). The first part, comprising this introductory section, serves to underscore the need for this manifesto. The second part offers a succinct overview of the debates on coastal governance and landscape governance, contextualising the imperative need for coastal landscape governance within the European context. Lastly, part three, the core of this article, presents and discusses the manifesto's seven recommendations to reframe the relationship between coastal and landscape governance towards coastal landscape governance.

## Contextualising coastal and landscape governance debates

### The coastal condition and the need for substantial reform in coastal governance

Coastal regions are dynamic and complex socio-ecological systems, characterised by intricate interactions amongst land, sea, and atmosphere (Bazant-Fabre et al. 2022). These regions hold significant ecological, cultural, aesthetic, social, and economic values (de Andrés et al. 2023). However, the process of coastalisation,^1^ prevalent in most coastal countries (Lagarias and Stratigea 2023), combined with the impacts of climate change (IPCC 2022), poses several threats to the integrity and governance of coastal regions.

Globally, intact coastal regions are becoming increasingly rare (Williams et al. 2022), a trend also observed in Europe. With 40% of the European Union’s population living near the coast and 75% of its foreign trade conducted by sea (European Environment Agency 2024), these regions face immense pressures from human activity. Furthermore, rising sea levels, extreme weather events, inundations, and aggravation of levels of erosion exacerbate the vulnerabilities of European coastal regions.

Indeed, despite five decades of Integrated Coastal Management efforts, critical shortcomings persist, prompting continued advocacy for integrated approaches from both the political and the scientific communities (Kelly et al. 2019). These shortcomings include overlapping political-administrative structures and actors, as well as challenges in multi-level governance (Eger et al. 2021). Fragmented and sectoral approaches prevail (Pérez-Cayeiro et al. 2019), with limited involvement of actors, particularly local coastal communities (Van Assche et al. 2020). Additionally, there is a lack of innovative mechanisms for policy integration, coordination, and cooperation (Eger et al. 2021), along with unstable policy discourses and shifting paradigms at a global scale without ex-ante or ex-post assessment of their impacts (Flannery and McAteer 2020). Furthermore, recent discussions highlight the absence of formal governance arrangements with specific actors^2^ and institutions^3^ recognising the coastal zone as a socio-ecological system within its specific governance arena while attending to its evolutionary governance pathway (Van Assche et al. 2020; de Andrés and Barragán-Muñoz 2022).

The severity of climate challenges in most European coastal countries requires a re-evaluation of how we conceptualise and operationalise coastal governance (de Koning et al. 2023). Such a re-evaluation encompasses not only the capacity of the coastal system to adapt and transform in response to new climate conditions but also the ability of individuals and organisational actors to drive systemic changes within society (Sousa et al. 2023). We argue that such an alteration must start with redefining how we conceptualise the coastal zone. Understanding the character and dynamics of the coastal system is fundamental for any significant shift. This shift involves embracing the socio-ecological boundaries and dynamics, incorporating the “coastal condition”, and recognising the interdependence of governance elements such as actors, institutions, power, knowledge, and narrative (Van Assche et al. 2014). Landscape governance can contribute to this paradigm shift, as will be discussed in the following sections.

### Landscape as a medium for coastal governance

Landscape governance is based on the concept of landscape, which entails a multi-sector, multi-actor, and multi-level process of decision-making and interaction (Van Oosten et al. 2021). However, the definition of landscape lacks consensus within scientific discourse, as it can vary depending on one’s scientific background (Kühne 2019). In this perspective, we adopt the formal definition of landscape as outlined in the Council of Europe Landscape Convention, which defines landscape as “*an area, as perceived by people, whose character is the result of the action and interaction of natural and/or human factors*” (Council of Europe 2000, 2016, article 1a). Similarly, landscape governance also does not has a universal definition as the debate is still evolving, yet its potential for success is widely acknowledged in landscape research (Gonçalves and Pinho 2022). This potential arises from its inherent scalability for intervention, integration of natural and cultural values, and capacity to provide common ground for various disciplines, sectors and stakeholders (Albrechts et al. 2020). Some scholars even argue that landscape governance is the spatialisation of environmental governance (Visseren-Hamakers 2015).

Coastal landscape governance builds on the conceptualisation of landscape governance, focussing specifically on the coastal landscape. The coastal landscape represents a unique landscape unit shaped by a particular combination of environmental, cultural, perceptual, and symbolic elements, as well as identifiable dynamics that set it apart from the rest of the territory (Nogué et al. 2016). Coastal landscapes are amongst the most fragile and dynamic types of landscapes due to the continuous interactions between land and sea, alongside their appeal for human activities, which subject them to unique pressures. Biophysical elements do not solely determine coastal landscapes. Coastal landscapes are also shaped by social, economic, and cultural relations established over time with their inhabitants, influencing their identity and character and making their governance more challenging (Egberts 2019; Schlüter et al. 2019). The coastal landscape is also a sensorial experience that can have multiple perceptions, meanings and values depending on the experiences and practises of those who are experiencing it, as demonstrated by Döring and Ratter (2021) research on the Wadden Sea.

It is imperative to conduct further research on coastal landscape governance due to its unique challenges compared to the governance of other landscapes. Coastal landscape governance entails managing both terrestrial and marine environments, which introduces governance complexities. These complexities involve addressing rapidly changing environmental conditions while also considering the needs, experiences, and perceptions of coastal communities. The significance of local coastal communities for coastal governance is increasingly emphasised in scientific discussions, particularly in light of climate change impacts (Van Assche et al. 2020; McKinley et al. 2022). Moreover, coastal landscape governance complexity includes navigating overlapping jurisdictional boundaries on land and at sea, balancing competing interests in land use—such as conservation, recreation, cultural values, and economic interests—meeting pluralistic goals, and integrating local priorities and knowledge into the governance system.

The diversity of conceptualisations of the landscape is also reflected in various methods for identifying and characterising coastal landscapes as they are rooted in a range of epistemological backgrounds and scientific disciplines (Antrop 2006; Simensen et al. 2018; Kühne 2019; Taylor and Xu 2019). This diversity of concepts and methods can lead to criticism regarding its potential to rethink coastal governance towards coastal landscape governance. However, like landscape, the coastal governance object also has no established and consensual concept (coastal zone, coastal area, littoral, coast, coastal socio-ecological system) or method to identify its boundaries (Kay and Alder 2005; Pittman and Armitage 2016; de Andrés and Barragán-Muñoz 2022). Also, the scientific demand for interdisciplinary and transdisciplinary approaches, building both from natural and social sciences to reinforce coastal governance (Partelow et al. 2023), along with the call for coastal governance to move beyond administrative boundaries (Van Assche et al. 2020), creates several opportunities for coastal landscape governance.

At the European level, these opportunities are reinforced by the existence of formal definitions of landscape (Antrop 2017), such as the one established by the Council of Europe Landscape Convention (Council of Europe 2000, 2016). The Convention also creates opportunities for landscape governance as it advocates for the establishment of a specific governance arena for landscape in its signatories-countries. Research is currently very limited on the connections between the Convention and scientific discussions on landscape governance. However, the review conducted by Pătru-Stupariu and Nita (2022) concerning the impacts of the Convention on interdisciplinary and transdisciplinary research indicates the emergence of landscape governance as a new study area in Europe. The review also expects continuous reinforcement of Landscape Sustainability Science^4^ as a research trend. This recognition underlines the importance of expanding research on the conceptualisation and operationalisation of landscape governance in Europe, and we claim that this is particularly relevant regarding coastal landscapes, as it was previously said, due to its significance for Europe and because they are especially susceptible to the impacts of climate change.

Furthermore, landscape governance is strongly related to discussions about Integrated Landscape Approaches (Mpofu et al. 2023), as it is the dominant approach to operationalising landscape governance in the Global South (Gonçalves and Pinho 2022). Reed et al. (2020, p. 2), building on the literature, describe these as the “*next generation of integrated approaches*”. We underscore the relevance of further research into the complementarities and contradictions regarding Integrated Coastal Zone Management or Marine Spatial Planning. Conceptually, Integrated Landscape Approaches possess two intrinsic characteristics that warrant exploration when compared with Integrated Coastal Zone Management or Marine Spatial Planning: first, they are grounded on the landscape concept, which means that they develop inclusive place-based policies/strategies, not limited by political-administrative boundaries, and second, they consider trade-offs as a critical premise for establishing management goals. Trade-off assessment is also considered a fundamental research area for improving coastal governance efficiency and effectiveness (Davies et al. 2018).

## A manifesto for coastal landscape governance

Some may consider the following arguments utopian, acknowledging the potential challenges in implementing coastal landscape governance. As Meisner (1982, p. 4) suggests, “*it is the striving for utopia, not its accomplishment, that is the dynamic force in history—and indeed a historically necessary one*”. Instead of waiting for a future time when civil society will demand change, possibly reaching a point where undoing the losses suffered becomes difficult, the appropriate moment to begin is now (Elrick-Barr et al. 2024). The following manifesto can serve as a guiding framework for a new European coastal landscape governance agenda, envisioning a roadmap for the future. However, we also argue that it is fundamental to increase empirical research on coastal landscape governance to validate its theoretical added value. We invite other researchers from other interdisciplinary and transdisciplinary perspectives to contribute to this research agenda.

### Beyond outstanding coastal landscapes: Coastal landscape governance addresses all landscapes and their evolution

In Europe, the conservation of nature and landscape began in the 1950s, primarily through the establishment of protected natural areas (Antrop 2013). The Council of Europe Landscape Convention represents a shift from early landscape conservation policies that were only focussed on protecting or enhancing exceptional coastal landscapes as crystallised objects (Antrop 2013). It emphasises the significance of ordinary landscapes for quality of life and asserts that Member States must legally recognise the landscape in the law (Council of Europe 2000, 2016). However, implementing a holistic landscape conceptualisation across most European countries remains challenging. Presently, there is still an emphasis on protecting remarkable coastal landscapes or approaching them purely as scenic landscapes within the legal framework of protected areas (Gonçalves and Pinho 2024; Rangel-Buitrago 2019). We contend that signatories-countries of the Council of Europe Landscape Convention must establish a governance system that considers all coastal landscapes, whether natural or cultural, urban or rural, marginal or central. This entails identifying what constitutes a coastal landscape not only at present but also understanding its evolutionary trajectory to adapt coastal landscape governance models to reflect their character across scales and timelines. But considering all coastal landscapes, a question arises whether they should be limited by the boundaries of coastal municipalities, regions, or countries.

### Beyond political-administrative boundaries: Coastal landscape governance recognises the coastal socio-ecological system boundaries and dynamics

In many European coastal countries, contemporary coastal governance is characterised by multiple political-administrative boundaries, often overlapping on land and at sea (de Andrés and Barragán-Muñoz 2022). The increasingly global and European focus on the blue economy and marine spatial planning (Rafael et al. 2024) further complicates coastal governance, highlighting the importance of land-sea interactions (Innocenti and Musco 2023). As discussed in “The coastal condition and the need for substantial reform in coastal governance” section, there is a growing call for the more effective integration of the coastal condition into the governance system while attending to its evolutionary pathway as a means to reinforce the adaptability of the governance system (Van Assche et al. 2020). Consequently, the debate on coastal governance underscores the necessity of recognising coastal zones from a socio-ecological perspective, transcending political-administrative boundaries (Schlüter et al. 2020; Van Assche et al. 2020; de Andrés and Barragán Muñoz 2022). This recognition also involves addressing land-sea interactions to adapt the governance system to handle coastal dynamics and their associated challenges.

Coastal landscape governance presents several opportunities to address these challenges as its governance system focuses on the coastal landscape. A coastal landscape is a concept rooted in landscape research that, despite its ontology and epistemology, is not limited by artificial boundaries. In Europe, as already stated, coastal landscape governance can be grounded in the formal concept of landscape promoted by the Council of Europe Landscape Convention (Council of Europe 2000). The Convention’s definition of landscape (see “Landscape as a medium for coastal governance” section) builds on a positivist and constructivist perspective. Besides assuming the coastal landscape as a socio-ecological system with a temporal-spatial scale, it also recognises it as an intangible, aesthetic and sensorial value, where democratic values are a central point (Antrop 2006; Egoz et al. 2018; Haller and Branca 2022). This holistic perspective of defining a coastal landscape can help to identify the coastal condition of a specific place, which is quite relevant as the signatory members of the Convention are demanded to identify and assess their landscapes throughout its territory, involving its citizens in this process from an evolutionary perspective (Council of Europe 2000, 2016). This obligation creates a formal opportunity for coastal landscape governance, as most European countries have signed and ratified the Convention. Furthermore, while the focus of this article may not extend to this particular aspect, it is relevant to note that since the 2016 amendment to the Convention, any country worldwide can sign it. This significant change effectively opens the doors to expanding coastal landscape governance beyond European borders.

A critique already mentioned in “Landscape as a medium for coastal governance” section is that various scientific perspectives and methods exist (Simensen et al. 2018) to identify a coastal landscape. However, we claim that this can be an opportunity to strengthen their governance, as the multiple meanings of landscape and methods can facilitate communication and cooperation between different areas of knowledge. Also, the juxtaposition of the different perceptions can lead to a more accurate perspective of the diverse viewpoints of the coastal socio-ecological system and the trade-offs necessary to reach a shared future vision. It also allows for the approach of power relations between the different stakeholders. Coastal landscape governance must, therefore, cultivate interdisciplinary and transdisciplinary knowledge spanning scientific disciplines, sectors, and policy domains to develop effective and efficient policies. For example, in the case of Portugal (Gonçalves and Pinho 2024), coastal landscape policies operate between coastal, environmental, landscape and spatial planning institutions and actors, being necessary to involve stakeholders with different and complementary expertise and imaginaries of the coastal landscape.

Another critical point is that the limits of what constitutes coastal landscape demand a re-evaluation of the landscape governance system to embrace its boundaries and dynamism into the different components of the governance system itself (Nogué and Sala 2018a). Despite the obligation to identify its landscape in the Convention’s signatories countries, in many coastal countries, coastal landscape units may lack the necessary legal framework to function effectively (Nogué and Sala 2018b). Some examples, such as those in Galicia, Spain, influenced by the Convention, have already utilised the concept of a landscape unit in developing their coastal zone management plans rather than confining it to a buffer area or administrative boundaries (García-García and Sanchiz 2017).

Further research is necessary to understand how coastal landscape governance has been occurring in Europe and to explore the necessary legal or institutional changes for integrating coastal landscape governance into the existing legal structures, as this knowledge is still unsufficient. Empirical evidence is fundamental to help researchers and policymakers tailor possible new configurations of coastal landscape governance. Even so, we must highlight that research on other complementary topics can also offer valuable insights as they approach governance beyond statutory frameworks. It is fundamental to explore relationships between these similar debates. For example, soft planning has been gaining relevance in Europe as a new planning and governance concept. Soft planning can be a helpful debate as it “*acts in complex, non-statutory institutional settings, breaking with established government structures, fixed administrative boundaries and rigid organisational frameworks*” (Cavaco and Costa 2020, p. 1). Another relevant discussion is centred on watershed-scale and transboundary governance, as their governance is also not limited by established legal political-administrative boundaries. For example, the use of the social-ecological watershed system for effective fisheries management, maintaining watershed resilience, is discussed by Nguyen et al. (2016). But, at what scale should the coastal landscape be governed?

### Beyond ad-hoc projects: The role of regional design in coastal landscape governance

Coastal governance operates across various scales, ranging from the global to the local scale. Nevertheless, the systematic review conducted by Gonçalves and Pinho (2022) revealed a predominant focus on its operationalisation at the local scale. This observation is further supported by Cabana et al. (2023), who also advocate for increased emphasis on the regional scale within coastal governance. Indeed, Neuman and Zonneveld (2018) assert that the regional scale serves as an effective intermediary between the local initiatives and the broader national and global impacts. This assertion resonates with the argument theorised by Wu (2013). In introducing the concept of Landscape Sustainability Science, Wu (2013) emphasises the need for place-based research on landscape and at regional scales. He claims that the “*regional scale requires planning and design to “optimise” the spatial pattern of different landscapes types*”(Wu 2013, p. 1017).

In the context of coastal landscape governance, we also recognise the regional scale as pivotal for developing effective governance systems. While it is relevant to have a vision of the relationship between all scales (from the global to the local), the regional scale provides a framework for delineating the boundaries of the coastal landscape, recognising its natural and cultural assets and addressing its interactions with national and global levels. Moreover, the regional scale also enables the development of coherent guidelines at the local level for project-specific aspects without compromising the larger scales required to encompass ecological, cultural, social, visual and aesthetic values that define the coastal condition.

Reflecting on the appropriate instruments to operationalise coastal landscape governance and on the argument of Wu (2013, p. 1017) that the regional scale demands planning and design, we assert the relevance of landscape-based regional design (Nijhuis et al. 2020). Landscape-based regional design, as originally conceptualised by McHarg (1969), is an interdisciplinary approach to ecological planning and design. Today, it incorporates knowledge from systems thinking and complexity theory to develop comprehensive regional planning and design strategies that prioritise the biophysical landscape structure and natural processes (Nijhuis et al. 2020) while also considering existing cultural, aesthetic, and sensory values. A landscape-based regional design allows for the development of open-ended strategies that seek a set of solutions for a coastal landscape, shaping new landscape imaginaries of coastal futures that guide and anticipate its development (Nogué and Wilbrand 2018). A landscape-based regional design strategy must visually illustrate how various stakeholder’s knowledge, interests and needs can be conciliated to formulate a cross-sectoral, place-based strategy tailored to a specific coastal landscape within its geographical, cultural, political and economic context. Landscape-based regional design also allows scientists, policy-makers, the private sector and civil society to explore alternative scenarios under uncertainty. This approach facilitates the integration of diverse perspectives and fosters collaborative decision-making processes, ensuring that coastal governance initiatives are inclusive, contextually relevant, and sustainable.

We also argue that landscape-based regional design must be developed using an Integrated Landscape Approach (Reed et al. 2020). An Integrated Landscape Approach demands continual learning, adaptive management, the identification of shared values, accounting of trade-offs on differing landscape uses, awareness of various governance levels, recognition of all relevant actors, transparency through mutual understanding amongst actors, clarity on rights and responsibilities of the actors, participatory monitoring while recognising different knowledge systems, resilience and enhancing the capacity of actors to engage (Mpofu et al. 2023). However, who are the ‘right’ stakeholders to be involved?

### Beyond “landscape elitism”: Coastal landscape governance entails landscape justice through knowledge co-production

In coastal governance, the participation of the 'right' stakeholders is crucial in light of the growing importance of knowledge co-production between researchers and non-academic actors (Celliers et al. 2023). The ‘right’ stakeholders “*must be able to act in some meaningful way*”, and for that, they need to have agency^5^ (Celliers et al. 2023, p. 1419). Coastal governance frequently fails to recognise and incorporate local communities’ knowledge, posing challenges in resource management amidst social-ecological shifts (Van Assche et al. 2020).

In landscape research, the idea of justice, facilitated through knowledge co-production, serves as a central pillar of the Council of Europe Landscape Convention (Kristensen and Primdahl 2020). The Convention advocates for the formulation of ‘landscape quality objectives^6^’, moving away from exclusive expert decision-making known as ‘landscape elitism’ (Scott 2011). Egoz and Nardi (2017) emphasise the importance of the ‘right to the landscape’ in enhancing the quality of life and in pursuing landscape justice. They assert that landscape justice supports landscape democracy (Gailing and Leibenath 2017; Egoz et al. 2018). Landscape democracy advocates for bottom-up approaches, recognising individuals as the foundation of any landscape and embracing diverse perspectives as a tool for social justice (Gailing and Leibenath 2017).

Coastal landscape governance embodies climate and landscape justice through knowledge co-production, emphasising the formulation of landscape quality objectives to envision and guide future regional strategies. These objectives must reflect the ‘right’ stakeholder perspectives (Celliers et al. 2023), including local coastal communities, fostering democratic and equitable processes. While not everyone may achieve their desired outcomes, discussions on future coastal visions are essential catalysts for social change and landscape justice. Reassessing power dynamics and institutional structures is imperative for producing political changes benefiting marginalised coastal communities. The Landscape Observatory of Catalonia (Spain) serves as an excellent example of involving local communities and stakeholders in defining landscape units and quality objectives through a centre that involves the nexus of research-practise-politics (Nogué et al. 2016). Indeed, the innovative approach of the Catalonia Government in establishing the Landscape Observatory of Catalonia to implement the Convention, underscores the feasibility and effectiveness of the political will in reshaping the landscape governance system. Nonetheless, given that not all regions and countries are like Catalonia (De Montis 2014), a question remains: how should the governance system be transformed towards coastal landscape governance?

### Beyond the absence of a specific actor: Coastal landscape governance demands a specific arena

Policy integration is one of the most significant challenges of coastal governance in the twenty-first century (Van Assche et al. 2020). The authors assert that coastal governance often faces challenges stemming from policy fragmentation, leading to inadequate policy integration and coordination amongst the various institutions and stakeholders responsible for planning and managing the coast. These challenges, Van Assche et al. (2020) argue, are further compounded by the limited understanding of the complex ecological processes and socio-economic dynamics that shape coastal landscapes.

In response to these challenges, Van Assche et al. (2020) advocate for the creation of a separate arena solely dedicated to coastal governance, designed with a specific focus on addressing the complexities intrinsic to the coastal condition. They highlight the need to embrace the coastal governance evolutionary pathway, including between land and sea, emphasising that only with a deep understanding of existing governance structures and integration mechanisms can researchers and policymakers propose the necessary structural and institutional changes fundamental to establishing this new governance arena.

Having a specific and tailored governance system is crucial, as in many European countries, coastal, environmental, landscape, and spatial planning operate under different governance systems, often resulting in conflicting interests, narratives, and power relations (Rodríguez-Rodríguez and Sinoga 2022). Moreover, as already stated, land-sea interactions, in most cases, also involve different actors with varying jurisdictions, responsibilities, and interests, thereby complicating multi-level governance (Kerr et al. 2015).

We also suggest that advancing coastal landscape governance requires establishing a dedicated arena with its institutions, and actors to approach the coastal landscape as a unifying spatial area with a long-term strategy. Establishing a dedicated coastal landscape governance arena can help mitigate impacts on the coastal socio-ecological system and manage various pressures.

It is fundamental to implement adaptive and integrated policies and ex-ante and post-assessment instruments to monitor changes in the coastal landscape over time. The responsibility for creating such a structure lies with the State, which should restructure the governance system according to its own form of government. The State must define the governing system, establishing principles, values, scales, institutions and actors that set the stage for planning and management approaches. This restructuring entails allocating the necessary institutional, financial, and human resources, as well as establishing the political and policy developments necessary to operationalise it. Coastal landscape governance should also encompass its relationship with the planning and management institutions by exploring the relevance of an Integrated Landscape Approach as a bridge between governance, planning and management.

### Beyond downsizing the state: Coastal landscape governance addresses the common good

Coastal policy trends in Europe have been changing without any assessment, replacing one paradigm with another without fully understanding their impact on the coastal condition (Gonçalves and Pinho 2024). Also, in many coastal countries, over the past decades, the State has downsized its role following market and neoliberal ideology (Tafon et al. 2021). In some cases, it has even privatised stretches of the coastline (Cabral and Alino 2011). This process often leads to inadequate resource allocation, limited policy development with a short-term focus, overexploitation of coastal resources and landscape degradation, which in turn increases the challenges in adapting to climate change.

As previously stated, coastal landscape governance involves an ongoing dialogue and consensus-building process with non-public actors and citizens through knowledge co-production. However, we also claim that the State must play the most significant role in governing and ensuring that the coastal landscape remains a common and public good (Ostrom 2015). The State must reassume control of coastal landscape infrastructure instead of subjecting public goods to the priorities of market investors. It is fundamental to make coastal landscape governance a political priority, raising awareness amongst policymakers about the importance and vulnerability of coastal landscapes. Citizens also play a fundamental role in demanding that their coastal landscapes be protected and managed sustainably, as the consequences of mismanagement directly impact or neglect them (Falaleeva et al. 2011). Debates on future visions for the coastal landscape are vital catalysts for social change away from market ideologies and interests. The State must create an ambitious shared vision to mobilise society to tackle the climate crisis and find sustainable solutions.

### Beyond a Euro-American theory: Regionalising coastal landscape governance

Landscape research is still mainly rooted in a Euro-American perspective (Antrop 2006, 2013). Nevertheless, it is worth noting that the empirical debates on landscape governance and Integrated Landscape Approaches have played a crucial role in addressing landscape forest restoration in the Global South. This evidence is primarily influenced by the involvement of various international organisations, such as the FAO, IUCN, GLF, and WWF, which have been instrumental in politicising these debates. Furthermore, also in the Global South, it is worth mentioning that the aspiration and scope of the Council of Europe Landscape Convention have significantly influenced the emergence of the Latin American Landscape Initiative and later its calls for an International Landscape Convention (Jorgensen 2017).

Despite this perspective mainly focuses on European coastal landscapes, we acknowledge the necessity to regionalise coastal landscape governance. Only a rigorous theoretical and empirical discussion built from the Global North and South, and from different interdisciplinary and transdisciplinary perspectives, will be able to build a coastal landscape governance theory that responds not only to European challenges but also to global governance challenges. Coastal landscape governance must engage with context-specificities and diversities around coastal landscapes and the local communities and actors that shape them to build a better and just landscape.

## Concluding remarks

There is an imperative need for transformative change in European coastal governance. Transformative change depends on its transformative capacity. By challenging established governance frameworks as suggested in this perspective, transformative capacity can be built/strengthened in order to catalyse a fundamental and systemic transformative change towards more sustainable pathways. Our manifesto, diagrammatically represented in Fig. 1, aims to catalyse and instigate a European research commitment to and through the governance of the coastal landscapes, acknowledging the limited scientific debate in this field. It also intends to elucidate its significance for the management of the coastal landscape, presenting several recommendations. We propose a paradigm shift towards coastal landscape governance, arguing for the recognition of coastal landscapes as unique socio-ecological systems and advocating for the establishment of dedicated governance arenas building on the Council of Europe Landscape Convention.

Our manifesto also emphasises the importance of considering all coastal landscapes, promoting landscape justice through knowledge co-production, and prioritising the common good over market interests. Furthermore, the manifesto calls for landscape-based regional design and highlights the need for a specific governance arena with inclusive and anticipatory governance approaches. Moreover, the manifesto also acknowledges the importance of regionalising coastal landscape governance, recognising the diverse perspectives and contextual specificities across different coastal regions that can contribute to this research agenda.

Our perspective serves as a call to action for researchers, policymakers, and stakeholders to collaborate in redefining coastal governance paradigms towards a more inclusive, adaptive, and justice-oriented framework. Landscape research can have a significant impact on driving this transformation, as it was demonstrated in Part Three of this article. By embracing coastal landscape governance, we can strive towards a future where coastal regions are sustainably managed, more resilient to environmental challenges, and more equitable for all stakeholders involved. While many doubts, questions or concerns may arise with our perspective, we considered that this research agenda and this call for change can serve as a starting point for action. It is essential to do so. Who is willing to join us?

## Footnotes

1 Coastalisation is a concept that refers to the rising concentration of population and activities in specific coastal zones (Lagarias and Stratigea 2023).

2 Actors are participants (individuals, groups or organisations) in governance, formally or informally (Beunen et al. 2015).

3 Institutions are rules (regulations, norms, laws, policies, and plans) of coordination between actors (Beunen et al. 2015).

4 “Landscape sustainability science is a place-based, use-inspired science of understanding and improving the dynamic relationship between ecosystem services and human well-being in changing landscapes under uncertainties arising from internal feedbacks and external disturbances” (Wu 2013, p. 999).

5 “Agency can be defined as the capacity of individuals and collective actors to change the course of events or the outcome of processes” (Celliers et al. 2023, p. 1419).

6 “The formulation by the competent public authorities of the aspirations of the public with regard to the landscape features of their surroundings” (Council of Europe, 2000, 2016, article 1c).
